# Evolutionary Conservation of the Ribosomal Biogenesis Factor Rbm19/Mrd1: Implications for Function

**DOI:** 10.1371/journal.pone.0043786

**Published:** 2012-09-12

**Authors:** Yvonne Kallberg, Åsa Segerstolpe, Fredrik Lackmann, Bengt Persson, Lars Wieslander

**Affiliations:** 1 Department of Molecular Biology and Functional Genomics, Stockholm University, Stockholm, Sweden; 2 Bioinformatics Infrastructure for Life Sciences and Swedish eScience Research Centre, IFM Bioinformatics, Linköping University, Linköping, Sweden; 3 Bioinformatics Infrastructure for Life Sciences, Science for Life Laboratory, Centre for Molecular Medicine, Karolinska Institutet, Stockholm, Sweden; 4 Science for Life Laboratory, Department of Cell and Molecular Biology, Karolinska Institutet, Stockholm, Sweden; Tel Aviv University, Israel

## Abstract

Ribosome biogenesis in eukaryotes requires coordinated folding and assembly of a pre-rRNA into sequential pre-rRNA-protein complexes in which chemical modifications and RNA cleavages occur. These processes require many small nucleolar RNAs (snoRNAs) and proteins. Rbm19/Mrd1 is one such protein that is built from multiple RNA-binding domains (RBDs). We find that Rbm19/Mrd1 with five RBDs is present in all branches of the eukaryotic phylogenetic tree, except in animals and Choanoflagellates, that instead have a version with six RBDs and Microsporidia which have a minimal Rbm19/Mrd1 protein with four RBDs. Rbm19/Mrd1 therefore evolved as a multi-RBD protein very early in eukaryotes. The linkers between the RBDs have conserved properties; they are disordered, except for linker 3, and position the RBDs at conserved relative distances from each other. All but one of the RBDs have conserved properties for RNA-binding and each RBD has a specific consensus sequence and a conserved position in the protein, suggesting a functionally important modular design. The patterns of evolutionary conservation provide information for experimental analyses of the function of Rbm19/Mrd1. *In vivo* mutational analysis confirmed that a highly conserved loop 5-β4-strand in RBD6 is essential for function.

## Introduction

The RNA-binding domain (RBD), also called RNA recognition motif (RRM), is present in a large number of proteins involved in various aspects of gene expression [1]. The RBD is present in prokaryotes [2], viruses and most abundantly in eukaryotes. For example, approximately 2% of mammalian proteins contain this domain [3]. In eukaryotes, one to six RBDs can be present in a single protein, often combined with other types of protein domains. The RBD is approximately 90 amino acid residues in length and has a typical topology consisting of a four-stranded β-sheet packed against two α-helices (reviewed in [4]). A basic RNA-binding mode has been described in which three aromatic rings, present in the conserved RNP1 and RNP2 motifs located in the β3 and β1 strands respectively, are important [5]. Other parts of the domain also contribute to binding specificity and affinity, giving the possibility to modulate the binding capacity. In addition, RBDs can interact with each other and with other protein domains, making the RBD a most versatile RNA- and protein-interaction domain [6].

Although RBD-containing proteins are found in all eukaryotes, there are many organism-specific RBD proteins and only three appear to be well conserved at the primary structure level in all studied eukaryotes; a nuclear cap binding protein, the poly(A) binding protein and the ribosome biogenesis protein Rbm19/Mrd1 [7].

In *Saccharomyces cerevisiae (S. cerevisiae)*, Mrd1 is essential for synthesis of small ribosomal subunits [8]. In the nucleolus, it associates cotranscriptionally with the pre-rRNA and, together with several other proteins and some snoRNPs, is required for pre-ribosomal maturation processes that lead to formation of pre-40S ribosomal subunits [9], [10]. Mrd1 contains five RBDs and connecting linker regions. In Mrd1, all five RBDs contribute to the function of the protein, but the individual RBDs are not of equal importance [9]. *In vivo* UV-cross-linking analyses show that Mrd1 is positioned in close proximity to the part of the 18S region within the pre-rRNA that will form the central pseudoknot in the mature 18S rRNA (Segerstolpe et al., manuscript in preparation). Based on experimental findings and the presence of multiple RBDs, it is likely that Mrd1 interacts with the pre-rRNA and possibly other proteins to take part in the formation of a processing competent pre-rRNP complex. The precise contribution of the different domains to the function of Mrd1 is not known.

Mrd1 homologues (names given in parenthesis after each species) have been studied in *Metarhizium acridum* (Mamrd1) [11], *Chaenorhabditis elegans* (RBD-1) [12], *Chironomus tentans* (RBD-1) [13], zebrafish (NPO) [14] and mouse (Rbm19) [15], [16]. The Mrd1 homologues are essential for cell growth, and in several cases they have been shown to be localized to the nucleolus [8], [15] and to be involved in 18S rRNA synthesis [11], [12], [13]. Studies in mice, homozygous for a gene-trap insertion in Rbm19, showed that Rbm19 is essential for formation of nucleoli during pre-implantation and that the protein may have functions in addition to pre-ribosome maturation [16].

Here we report an analysis of the degree of evolutionary conservation throughout eukarya of the domains and linkers in Rbm19/Mrd1. We find that the individual RBDs have specific positions and distances in relation to each other and that they have specific patterns of conserved residues. Experiments performed show that one of the defined conserved elements is essential for function of the protein. The pattern of conservation is likely to reflect structurally and functionally important elements in the protein.

## Results

### Two dominating conserved domain structures of Rbm19/Mrd1

**Figure 1:**
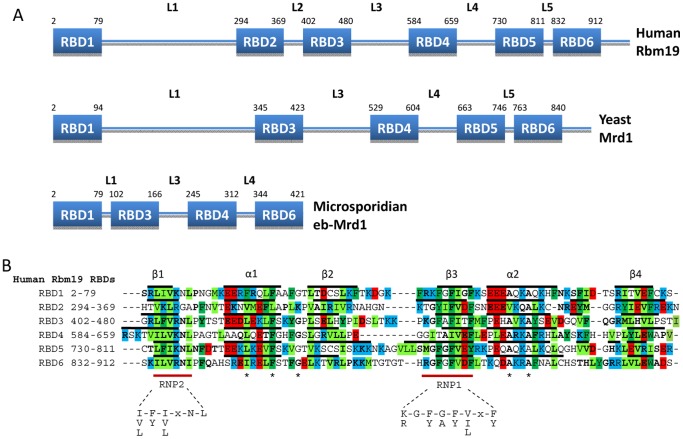
Domain organization of Rbm19/Mrd1. A. Human Rbm19, *S. cerevisiae* Mrd1 and *E. bieneusi* Mrd1 (eb-Mrd1) represent the three versions of the protein that are present in eukaryotes, having 6 (Rbm19), 5 (Mrd1) and 4 (eb-Mrd1) RBDs. The RBDs are numbered RBDs 1–6, and the regions connecting the RBDs are named linkers 1–5 (L1–L5), with the number corresponding to that of the preceeding RBD. B. Alignment of RBDs 1–6 in Rbm19 (human). Bold black lines above each sequence indicate the secondary structure elements (β-strands and α-helices). For RBD1 the secondary structure was predicted using Psipred [41]. For RBD2–6, secondary structure elements were extracted from the NMR determined structures with PDB identifiers 2DGW (human), 1WHW (mouse), 1WHX (mouse), 2CPF (mouse) and 2CPH (mouse), respectively. Residue properties are shown by background colour (blue = positively charged, red = negatively charged, green = hydrophobic). Residues in bold indicate participation in the consensus sequences identified in this paper. The conserved RNP1 and RNP2 motifs are indicated by the red lines below the alignment, as well as other generally conserved positions (asterisks).

In eukaryotes, Rbm19/Mrd1 homologues characteristically contain multiple RBDs. The human Rbm19 and the yeast Mrd1 proteins represent the two common versions of domain organization, consisting of six or five RBDs respectively (Fig. 1A). Here, these RBDs are numbered RBDs 1–6, starting from the N-terminus, based on Rbm19. As shown below, Mrd1 lacks RBD2. Linker regions, called linker 1–5, connect the RBDs. In addition, a short C-terminal extension is present. A minimal version of the protein, containing only four RBDs, is present in Microsporidia (Fig. 1A). Microsporidia belong to fungi and are spore-forming unicellular intracellular parasites in animals. As shown for Rbm19 in Fig. 1B, the RBDs conform to the characteristics of RBDs [1]. All six RBDs have four β-strands and two α-helices in the characteristic β1-α1-β2-β3-α2-β4 topology. Each RBD in Rbm19 has an RNP1 motif in the β3 strand, with aromatic residues at position 3 and 5, with the exception of RBD4 that lacks both these aromatic residues. All RBDs also have the less well-conserved RNP2 motif in the β1 strand, but only RBD3 and RBD5 have an aromatic residue at position 2 in this motif.

Apart from the RNP1 and 2 motifs, the six RBDs have different primary structures. However, several positions are conserved in RBDs in general. These include hydrophobic residues L/I/V/F and F/L in α1, a frequently conserved glycine in loop 2 (between α1 and β2) and most often two alanines in α2 (marked by asterisks in Fig. 1B) [17]. Most of these residues are located in a hydrophobic core of the RBD and presumably contributing to the RBD fold [18]. As seen in many RBDs, loop 3 (between β2 and β3) varies in length more than other parts of the RBD. These general features are also shared by the five RBDs in Mrd1.

We conclude that Rbm19 and Mrd1 have a common domain topology, but that the number of RBDs differs by one.

### Rbm19 and Mrd1 homologues are present in all eukaryotic organisms

**Figure 2:**
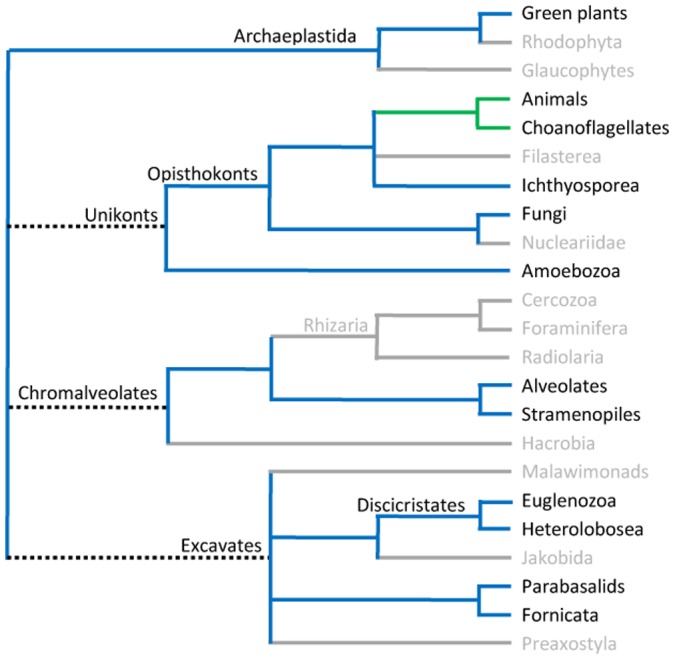
Phylogenetic tree of eukaryotic lineages showing Rbm19/Mrd1 proteins containing five or six RBDs. The tree is redrawn from http://tolweb.og/Eukaryotes/. Blue lines indicate organisms with five RBDs and green lines those with six RBDs. Grey lines indicate branches for which no completely sequenced genome is known yet. Dashed lines indicate uncertainties in the tree topology. Microsporidia are fungi, but exceptionally have only four RBDs.

No Rbm19/Mrd1 homologues could be detected in archaea or prokarya. We analysed the extent to which Rbm19/Mrd1 homologues are present in eukaryotes (Fig. 2). Furthermore, we investigated how species with proteins containing five or six RBDs are distributed in a eukaryotic phylogenetic tree. We observed that Rbm19/Mrd1 proteins that contain six RBDs are restricted to animals and Choanoflagellates, while all other branches for which reliable data were found, contain five RBDs (Fig. 2). The simplest explanation for this distribution is that RBD2 was introduced into an Rbm19/MRD1 gene in an ancestor common to animals and Choanoflagellates.

The widespread distribution of Rbm19/Mrd1 proteins in the eukaryotic tree argues that such a protein is present in all eukaryotic species and hence should have appeared very early in eukaryotic evolution. This is compatible with the hypothesis that Rbm19/Mrd1 is part of ribosome biogenesis, an essential and highly conserved process.

### Microsporidian genomes have a minimal sized Rbm19/Mrd1 homologue

**Figure 3:**
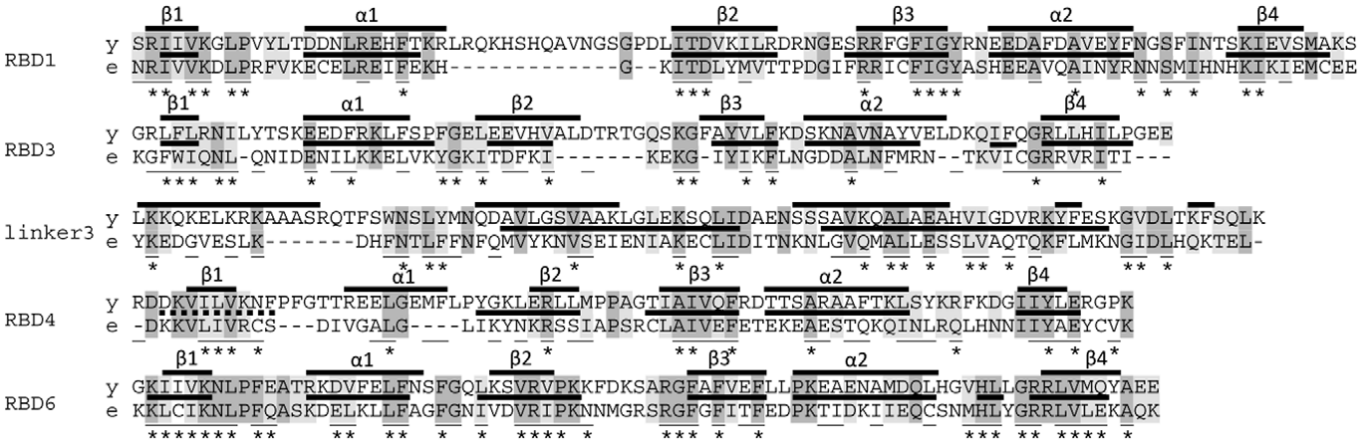
Alignment of the microsporidia Rbm19/Mrd1 homologues to *S. cerevisiae* Mrd1. Alignment of RBDs 1, 3, 4, 6 and linker 3 of *S. cerevisiae* Mrd1 (denoted y) to *E. bieneusi* Mrd1 (denoted e). Identical residues (dark grey) or similar (light grey) between the two homologues are indicated. Secondary structure predictions are shown above the sequences. Positions present in the general consensus sequences (see Fig. 5A) are underlined and asterisks indicate where 2 out of 3 of the microsporidia homologues (B7XJ60, C4V7E1 and E0S816) are conserved. Q8SRD9 was excluded due to high sequence similarity to E0S816 (>80% in the RBDs), in order to avoid bias.

The intracellular parasites of the Microsporidium family have a highly condensed minimal genome and generally have only approximately one third of the ORFs present in *S. cerevisiae* [19]. We found candidate Rbm19/Mrd1 homologous proteins in the four available complete genomes of these species (*Enterocytozoon bieneusi* B7XI60, *Encephalitozoon cuniculi* Q8SRD9, *Nosema ceranae* C4V7E1 and *Encephalitozoon intestinalis* E0S8I6). The proteins are unusually small, only around 420 amino acid residues long. The overall pairwise sequence identities among the Microsporidian Rbm19/Mrd1 homologues are typically 35–45%, with the exception of *E. intestinalis* and *E. cuniculi* which have 79% pairwise sequence identity towards each other. Microsporidia are fungi and although fungi in general have Rbm19/Mrd1 homologues with five RBDs (Fig. 2), the microsporidia proteins only show four RBDs in alignment with the other Rbm19/Mrd1 homologues, corresponding to RBDs 1, 3, 4 and 6 (Fig. 1A). The Microsporidian RBDs 1, 3 and 6 (Fig. 3, shown for *E. bieneusi*) are all predicted to fold into the common RBD topology, but in RBD4, β1 may not form since some predictions (*E. bieneusi* and *E. cuniculi*) suggest folding of this region into an α-helix. In addition, the microsporidia proteins have an unusually short linker 1 (median 16 residues, range 16–22) and linker 4 (median 25 residues, range 22–33). Linker 3 is similar in length to the Rbm19/Mrd1 linker 3, median of 81 residues (range 80–82), in comparison with 106 residues in Rbm19 and 108 residues in Mrd1. It also contains the linker 3 sequence motif (Fig. 3 and in the section about the linker regions).

Microsporidian RBD1 and RBD6 are highly conserved and easily identifiable. Both the RBD1-specific HMM (RBD1-specific hidden Markov model) and RBD6-specific HMM (RBD6-specific hidden Markov model) recognized E0S8I6 (*E. intestinalis*), Q8SRD9 (*E. cuniculi*) and B7XI60 (*E. bieneusi*), while only the RBD6-specific HMM was able to recognize C4V7E1 (*N. ceranae*). The microsporidia RBD1 and RBD6 showed convincing alignments to the consensus sequence of RBDs 1 and 6 (Fig. 3). RBD1 and RBD6 are also similar in sequence to the *S. cerevisiae* Mrd1 protein (Fig. 3, gray shading). RBD3 and RBD4 are less well conserved (Fig. 3, RBD3 and RBD4), and HMMs trained to find RBD3 and RBD4 did not recognize the corresponding microsporidia RBDs. However, the alignment shows that among the consensus positions, 53% and 42% of the positions are conserved in RBD3 and RBD4, respectively, in at least 2 of 3 microsporidia (Fig. 3), supporting a distant relationship.

Finally, we performed pair-wise sequence comparisons (using fasta34) of the four Microsporidian proteins versus UniprotKB. Comparing full-length sequences, as well as the parts corresponding to RBD1 and RBD6, all had Mrd1 homologues among the top list, above any other RBD-protein (data not shown). Only a very distant relationship to other, non-Rbm19/Mrd1, RBD-proteins was detected. Comparisons for RBD3 and RBD4 displayed uncertainties as to which genes these Microsporidian domains are closest to.

We conclude that Microsporidian genomes contain an Rbm19/Mrd1 homologue with reduced number of RBDs, linkers of reduced length and well-conserved RBDs 1 and 6.

### Position-specific conservation of RBDs

**Figure 4:**
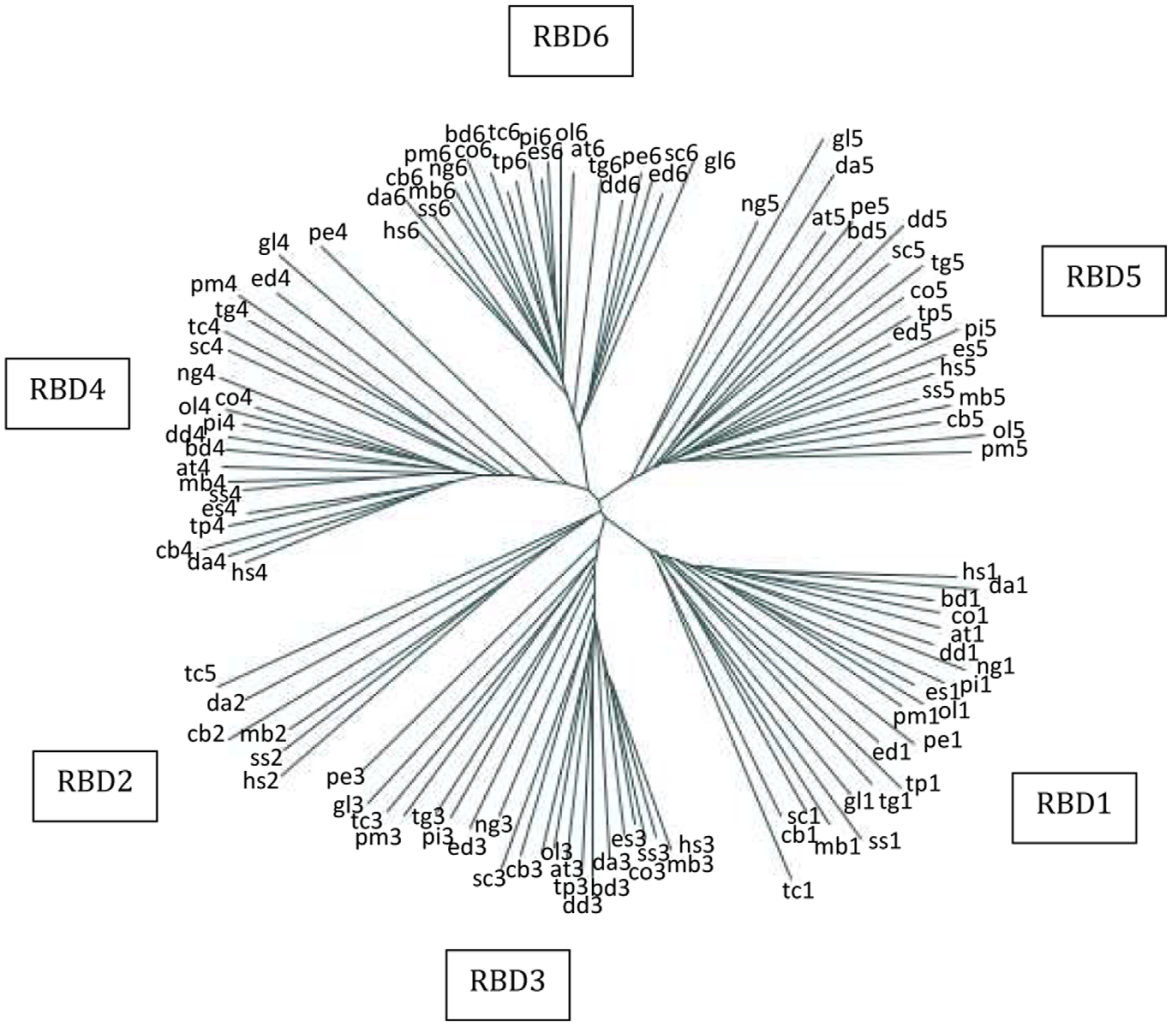
Dendrogram showing relationships between the RBDs. The six different RBDs form six clearly separated clusters, of which RBDs 1, 5 and 6 are most easily discernible. Each RBD is denoted with a two-letter code for species and a digit for the RBD number. Sequences are taken from: hs – *Homo sapiens* (Q9Y4C8), da – *Drosophila ananassae* (B3MYP1), ss – *Salpingoeca sp* (F2U536), mb – *Monosiga brevicollis* (A9USE7), cb – *Caenorhabditis briggsae* (A8WV73), bd – *Batrachochytrium dendrobatidis* (F4NSW1), dd – *Dictyostelium discoideum* (Q54PB2), tp – *Thalassiosira pseudonana* (B8BZC4), pi – *Phytophthora infestans* (D0NJ71), es – *Ectocarpus siliculosus* (D8LH81), at – *Arabidopsis thaliana* (F4JT92), ol – *Ostreococcus lucimarinus* (A4RVV1), tg – *Toxoplasma gondii* (B6KPW8), pm – *Perkinsus marinus* (C5KH14), sc – *Saccharomyces cerevisiae* (Q06106), pe – *Paramecium tetraurelia* (A0DWV5), ed – *Entamoeba dispar* (B0ECZ6), tc – *Trypanosoma cruzi* (E7KXH4), ng – *Naegleria gruberi* (D2V9G7), co – *Capsaspora owczarzaki* (E9C5E6), gl – *Giardia intestinalis* (A8BKE6). The number after each abbreviation indicates the RBD position.

Although all the RBDs in Rbm19 and Mrd1 have general characteristics of RBDs, the individual RBDs within each protein are different (Fig. 1B). In order to better understand these differences, we constructed a dendrogram for the individual RBDs present in Rbm19/Mrd1 homologues, using evolutionary representative full-length sequences, corresponding to those in Fig. 2 and one level below. Fig. 4 shows that the individual RBDs cluster in a specific pattern, showing that each RBD is conserved as to sequence and position within the protein. RBD1 and RBD6 were the most clearly grouped. We observed two exceptions to this pattern. First, in euglenozoa kinetoplastidia, RBD5 does not cluster with the corresponding RBDs in the other species (c.f. tc5 in Fig. 3), and the RBD5-specific HMM is not able to recognize them. Furthermore, linker 5 is exceptionally long (about 60–70 residues, see section about linker regions). RBD5 and linker 5 are very similar in ten different species belonging to euglenozoa kinetoplastidia, suggesting that sequencing errors are not involved. Furthermore, no introns have been reported in the corresponding gene in these species, so splice variants are not likely. Their RBD5 lacks a typical RNP1 motif and is not more similar to any RBD in Rbm19/Mrd1 than it is to RBDs in other proteins. Therefore RBD5 in these species is different. In Fig. 4, the *Trypanosoma cruzi* RBD5 (tc5) probably clusters with the RBD2 group because the other groups are more similar within the respective groups while the RBD2 group is more heterogeneous. Second, while RBD1 and RBD6 from Microsporidians did cluster as expected, RBD3 and RBD4 did not convincingly group with other RBD3 and RBD4 (data not shown).

We conclude from the phylogenetic analyses that an Rbm19/Mrd1 homologue with multiple RBDs is present in all 271 eukaryotes analysed, representing most eukaryotic evolutionary branches. The gene for Rbm19/Mrd1, including multiple RBDs with position-specific properties, should therefore have evolved very early during eukaryotic evolution. The distribution of proteins with five or six RBDs suggests that RBD2 appeared later than the other RBDs and that this only occurred in a subset of higher eukaryotes. Sequence comparisons of the different RBDs are not informative regarding the origin of RBD2. The Microsporidian Rbm19/Mrd1 homologues have apparently lost one RBD and linker sequences.

### Conservation of the individual RBDs

The RBDs are conserved as to sequence and position in Rbm19/Mrd1 (Fig. 4). Each of the six RBDs was therefore compared in more detail between Rbm19/Mrd1 homologues from available species (79–108 species, except RBD2 with only 22 species after 80% non-redundant filtration).

**Figure 5:**
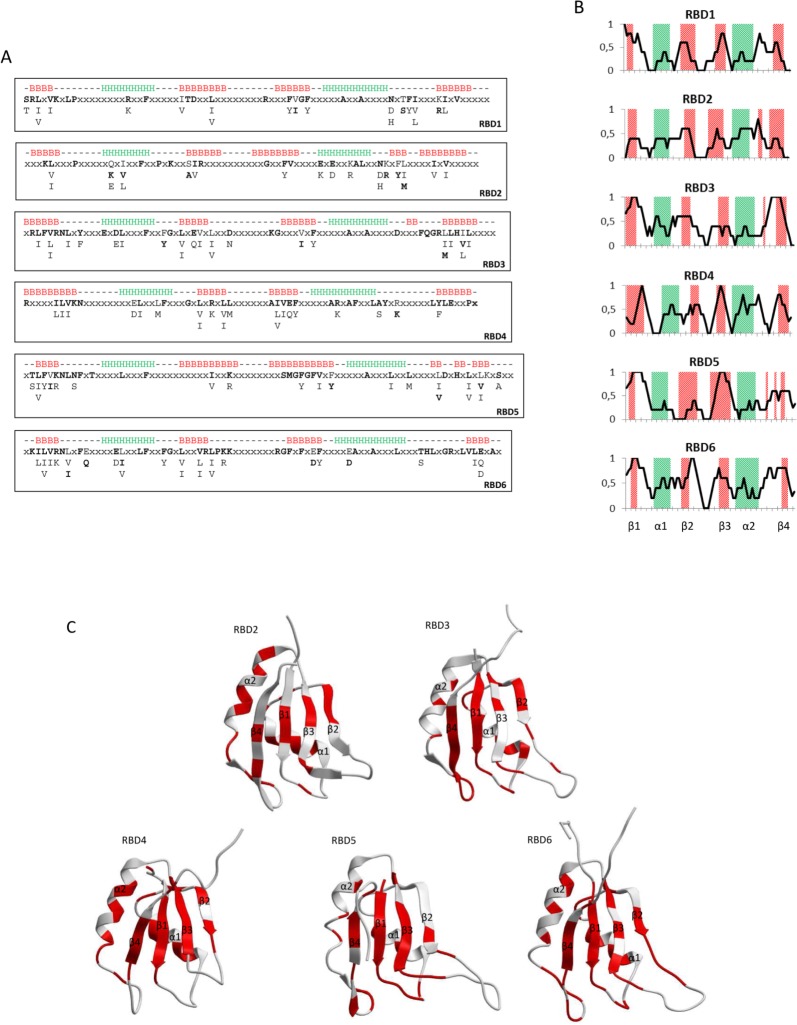
Sequence conservation characteristics of the individual RBDs. A. Consensus sequences of RBDs 1–6. The conserved residues are ordered according to frequency, with the most frequently occurring amino acid residue at the top. Rbm19 (human) residues are shown in bold. Secondary structure elements are derived as in Fig. 1B. B. Extent of conservation along each RBD. A window of five residues was slided along each of the six consensus RBDs, calculating the average presence of conserved residues (0–1, y-axis). This value is assigned to the central position of the window (solid line). The positions of α-helices are indicated in green and β-strands in red. C. Conserved residues in the 3D-structures of RBD2–6. Ribbon diagrams showing the 3D-structures of the human RBD2 (PDB identifier 2DGW) and the mouse RBD3–6 (PDB identifiers 1WHW, 1WHX, 2CPF and 2CPH, respectively), all in the same orientation, facing the β-sheet and with loops 1, 3 and 5 pointing downwards. Conserved residues are shown in red. In each RBD, the α1- and α2-helices as well as the β-strands (β1–β4) are labelled.

The overall conservation of the RBDs ranged from 33 to 52% (conserved positions/total positions: 27/78 (35%) in RBD1, 25/76 (33%) in RBD2, 35/79 (44%) in RBD3, 33/76 (43%) in RBD4, 32/82 (39%) in RBD5 and 42/81 (52%) in RBD6). The consensus sequences (Fig. 5A) are likely to represent structurally and functionally important features for each RBD. In Fig. 5B, the distribution of the conserved positions is shown along each RBD, demonstrating the unique pattern of conservation for each RBD. In Fig. 5C, the conserved positions are highlighted in the previously determined 3D structures of RBD2 in human Rbm19 and RBDs 3–6 in mouse Rbm19 (see PDB identifiers in figure legend to Fig. 5C).

For RBD1, no structure has so far been determined, but according to secondary structure predictions (Fig. 1B), it is most likely folded similar to other RBDs. The β-sheet is well conserved (Fig. 5B). The RNP1 motif has 5 out of 8 consensus residues, including position 1 and 5. The RNP2 motif has 4 out of 6 consensus residues, but lacks an aromatic residue at position 2. RBD1 has a specific, conserved glycine at position 7 in RNP1 and a conserved lysine at RNP2 position 4. Several conserved residues are present in β2 and β4. α1 contains a positive residue, but its orientation is not known. Loop 1 has one conserved residue and loop 5 is extensively conserved, containing two polar, one aromatic and one hydrophobic position. RBD1 is therefore characterized by a conserved β-sheet and loop 5 (Fig. 5B).

RBD2 has a less conserved β-sheet. The RNP1 and RNP2 motifs are weak; RNP1 contains 4 out of 8 consensus residues and RNP2 has 2 out of 6 consensus residues and only one residue critical for RNA binding is present (in RNP1 at position 5). α1 contains one conserved position with a polar or charged residue with a side chain pointing outwards. In α2 there are three charged positions. The first and last of these residues point outwards whereas the middle forms a hydrogen bond with a conserved lysine in loop 2 in the 3D structure. Conserved residues are found in loop 1 (one) and loop 2 (two). Loop 5, containing a short extra β-strand, has a cluster of conserved residues. It should be noted that for RBD2, there are fewer sequences available for analyses and that these sequences are present in species relatively closely related to each other (Fig. 2).

RBD3 has a conserved β-sheet. The RNP1 motif has 5 out of 8 consensus residues, including conserved position 1, but lacks aromatic residues. The RNP2 motif is fully conserved (6 out of 6 consensus positions). RBD3 has a specific, conserved arginine at RNP2 position 4. β2 and β4 have several conserved residues. α1 contains two negatively charged residues with side chains pointing outwards. Loop 1 and 2 have one conserved aromatic residue each and loop 3 has one conserved residue either charged or polar. Loop 5 stands out because it has an extra, short, β-strand and a cluster of conserved residues extending well into β4.

RBD4 has a conserved β-sheet, but poor consensus RNP1 (3 out of 8 consensus positions) and RNP2 (4 out of 6 consensus positions) motifs, lacking all three critical aromatic residues and a positive position 1 in RNP1. RBD4 has a conserved negatively charged residue at position 7 in RNP1 and lysine at position 4 in RNP2. α1 has one negatively charged residue pointing outwards. α2 contains a conserved aromatic residue, potentially interacting with residues in β1 and β4, and a positively charged residue, pointing outwards. Loop 5 has a conserved beginning, notably with an aromatic and a positive residue. A conserved stretch of four residues, including an aromatic position, extends from the end of loop 5 into β4.

RBD5 has a well-conserved β-sheet, except β2. The RNP1 motif is almost fully conserved (7 out of 8 consensus positions) and the RNP2 motif is completely conserved (6 out of 6 consensus positions). RBD5 has a conserved, positively charged lysine at RNP2 position 4. α1 and α2 have no conserved charged residues. As in RBD3 and RBD6, a conserved aromatic residue is present in loop 1. Apart from the β1- and β3-strands, the best-conserved part of RBD5 is the loop 5 - β4 regions. Loop 5 contains two short β-strands and several conserved residues are found here and in β4.

RBD6 is the overall most well conserved RBD. The RNP1 and RNP2 motifs are conserved (6 out of 8 and 5 out of 6 consensus positions, respectively), but an aromatic residue is lacking at position 2 in RNP2. RBD6 has a specific, conserved negatively charged residue at position 7 in RNP1 and a positively charged arginine at RNP2 position 4. Both α1 and α2 contain a negatively charged residue and both point outwards. Loops 1, 2, 3 and 5 all contain conserved residues. Loops 1 and 2 both include an aromatic residue. Loop 1 also has a negatively charged residue. Loop 3 is well conserved at its beginning where it has two positively charged residues. The end of RBD6 is very well conserved with the majority of loop 5 and β4 being conserved. Conserved residues are present at the penultimate position in β4 and the first position after β4, positions that are known to influence the specificity in the binding of a dinucleotide in the RNA by the central β1 and β3 strands (reviewed in [5]). A similar situation could be true for RBD5. Together with RBDs 3 and 5, RBD6 have conserved loops (loops 1, 3 and 5) that are all at the same side of the domain (Fig. 5C), with the conserved parts tending to be close to each other.

### Experimental confirmation of the functional importance of the conserved loop 5-β4 in RBD6

**Figure 6:**
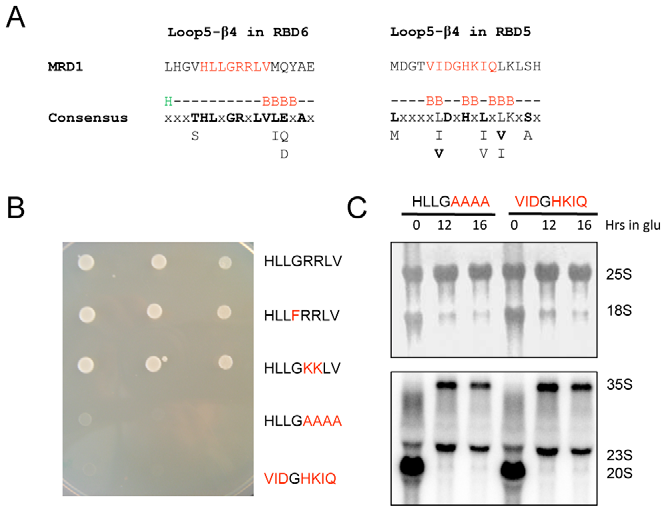
Mutational analysis of loop 5-β4 in RBD6 of Mrd1. A. The amino acid sequence of loop 5-β4 is shown for RBD6 and RBD5 of Mrd1 in comparison with the corresponding consensus sequences and secondary structure predictions. The analysed amino acid residues are shown in red. B. Growth characteristics of mutant *S. cerevisiae* cells compared to wild type. A dilution series (from left to right) of each mutant cell was spotted onto a selective FAA agar plate. The relevant amino acid sequence for each strain is shown to the right. Residues in red indicate the tested amino acid substitutions. The last mutant strain has the RBD6 sequence exchanged for the corresponding RBD5 sequence. C. Northern blot analysis of rRNA and pre-rRNA in the two mutant cells with impaired growth. The wild type *MRD1* gene under the control of a GAL promoter was shut off by growth in glucose medium for the indicated number of hours. Top panel shows the levels of 25S and 18S rRNA are shown after methylene blue staining of the membrane. Bottom panel shows the levels of the 35S, 23S and 20S pre-rRNAs are shown after hybridization with an oligonucleotide probe.

RBD6 is the most well conserved RBD in Rbm19/Mrd1 (Fig. 5A–C). One especially conserved region lies within the loop 5-β4-region, where 10 out of 13 residues are conserved (Fig. 5A and Fig. 6A). In order to test whether this region plays an important role, we used *S. cerevisiae* and mutated a region corresponding to amino acids 827–834 (HLLGRRLV) in Mrd1 (Fig. 6A). We substituted the glycine at position 830 to a phenylalanine (G830F). We also substituted two positively charged arginines into two positively charged lysines (RR831–832KK). We changed the RRLV region into four alanines (RRLV831–834AAAA) and replaced the whole region HLLGRRLV with the corresponding region of RBD5 (Fig. 6A), amino acid positions 733–740 (VIDGHKIQ). This resulted in the mutant RBD6(827–834)-RBD5(733–740).

To test for the ability of the mutants to support growth, the haploid cells, with the mutated region within the genomic *MRD1* allele, were spotted in a dilution series onto FAA plates where the cells were forced to lose the *TRP1* wt *MRD1* plasmid (Fig. 6B). The G830F mutation and the RR831–832KK mutation did not result in any growth defects as compared to wt cells (Fig. 6B). When the RRLV region was changed into alanines or the corresponding RBD5 region replaced the whole region, the mutations were lethal (Fig. 6B).

To further analyse the importance of the conserved loop 5-β4 region within RBD6 we constructed diploid strains with the lethal mutations (RRLV831–834AAAA and RBD6(827–834)-RBD5(733–740) in a strain background where the other wt *MRD1* allele could be conditionally shut off in glucose (*GAL1-3HA-MRD1*). We depleted the strains for wt Mrd1 during the times indicated and analysed rRNA and pre-rRNA (Fig. 6C). After 12 hours in glucose the amount of 18S rRNA was extensively decreased, whereas the steady state level of 25S rRNA was unaffected. Hybridization with a probe localised at the 3′ end of the 20S pre-rRNA region (between the D cleavage site that defines the 3′ end of the 18S rRNA and the A_2_ cleavage site that defines the 3′ end of the 20S pre-rRNA) showed that 20S pre-rRNA was reduced in the mutant cells when wt Mrd1 was depleted. This resulted in an accumulation of 35S pre-rRNA and 23S rRNA in the mutant cells due to lack of cleavage at A_0_–A_2_.

We conclude that the highly conserved loop 5-β4 region within RBD6 is essential for the function of RBD6 in *S. cerevisiae*. The growth defect was due to the inability of the mutant proteins to assist in the A_0_–A_2_ pre-rRNA processing, leading to a concomitant loss of 20S pre-rRNA and 18S rRNA. Our amino acid substitutions showed that the conserved glycine in the loop 5-β4 region is not essential, but suggest that two juxtapositioned positively charged residues are functionally important. The first positively charged residue is conserved (see consensus, Fig. 6A).

### The linker regions

We analysed the properties of the non-RBD regions (linkers 1–5 and the C-terminal extension) in 117 species in which the domain organization was clearly defined. These species represented metazoa, fungi, alveolata, amoebozoa, choanoflagellida, euglenozoa, heterolobosea, ichtyosphorea, stramenopiles and viridiplantae. The total length of the protein varied in these species between 697 and 2006 residues. The proteins had either four or five linkers (see Fig. 1A). In addition, the N-terminus preceding RBD1 generally was very short, typically 2–4 residues (but longer versions exist especially in alveolata), and the C-terminal extension following RBD6 was typically 30–60 residues, range 9–68.

In all species, the length relationships between the linkers were the same with a common order of decreasing length; L1 (median of 218, range 92–339), L3 (median of 101, range 96–183), L4 (median of 46, range 24–130), L2 (median of 32, range 23–42), L5 (median of 19, range 6–56), placing the RBDs at conserved distances from each other.

**Figure 7:**
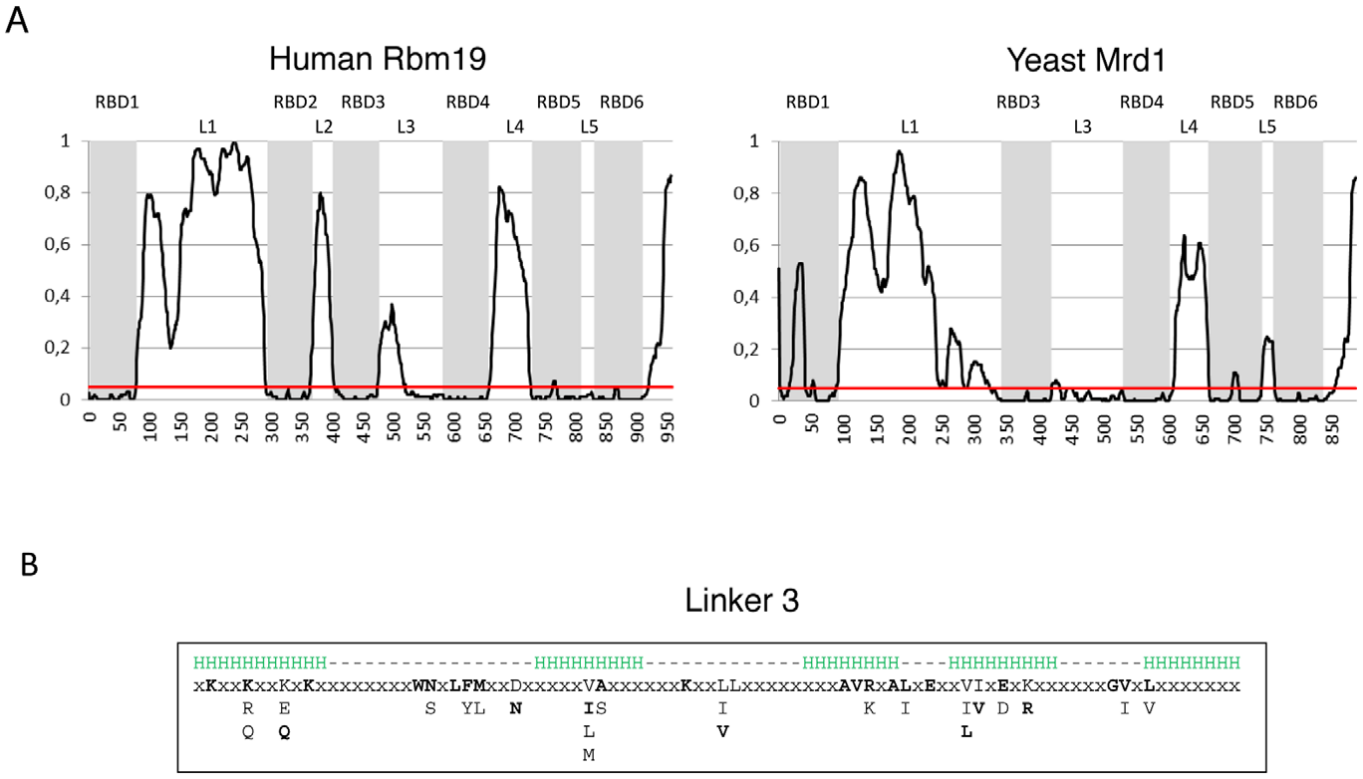
Properties of the linker regions. A. Disorder prediction of Rbm19 (human) and Mrd1 (*S. cerevisiae*). Grey areas indicate the RBDs: the red line represents the 0.05 threshold above which values are considered to indicate disorder. RBDs (RBD1–6) and linkers (L1–5) are indicated. B. Consensus sequence of linker 3. Conserved residues are ordered according to frequency, with the most common residue at the top. Rbm19 (human) residues are in bold. The secondary structure prediction given above the sequence (H = α-helix) is for linker 3 in Rbm19 (human).

A consensus sequence for linkers 1, 2, 4 and 5 could not be detected. Linkers 1, 2 and 4 are clearly disordered in most species according to predictions (Fig. 7A). Furthermore, linker 5 is in most cases disordered as well. Linker 3 is generally not disordered to the same extent as the other linkers according to predictions, although the first half can be (see Rbm19 in Fig. 7A). The sequence of linker 3 is also more conserved than those of the other linkers and a consensus sequence for linker 3 is observed (Fig. 7B). In many species, secondary structure predictions for linker 3 consistently indicate the presence of 4–5 α-helices (Fig. 7B), suggesting that linker 3 is structured. Conserved positions are distributed throughout the linker, although they cluster in the first helix, between the two first helices and at the second and third helix.

In conclusion, the linkers have a conserved length relation, positioning the RBDs at conserved distances from each other. The linker that varies most in length is linker 1. The amino acid sequences of linkers 1, 2, 4 and 5 are not conserved and these linkers are predicted to be disordered, while linker 3 appears to be structured and has several conserved residues.

## Discussion

### Origin of the modular architecture of Rbm19/Mrd1

An Rbm19/Mrd1 protein with a conserved modular architecture is present in all branches of the eukaryotic evolutionary tree (Fig. 2), indicating that this protein is involved in fundamental cellular process(es). In agreement, both versions with five and six RBDs, are essential for synthesis of the small ribosome subunit [8], [11], [12], [13]. From the distribution of the two most common variants of Rbm19/Mrd1 in the eukaryotic tree (Fig. 2), we assume that a protein homologue with five RBDs was present very early during the evolution of eukaryotes. The protein is not present in prokarya and archaea in which ribosome synthesis requires many fewer biogenesis factors than in eukaryotes (discussed in [20]). Rbm19/Mrd1 therefore seems to be coupled to evolution of a common eukaryotic way of synthesizing ribosomes.

A likely scenario is that the origin of Rbm19/Mrd1 was an RBD-containing protein present in an early eukaryotic ancestor. Duplication of domains within a protein coding gene is a common feature of evolution of RNA-binding proteins [21]. The five RBDs in the Rbm19/Mrd1 ancestor must have appeared through duplications and acquired their functional individuality very early (Fig. 4). In prokarya and archaea, RBD-containing proteins exist, but they are few and most proteins have only one RBD [2], [22]. Two prokaryotic 30S assembly factors, RbfA and Era [23], [24], contain one RNA-binding KH signature domain each and associate with the pre-ribosome at sites within the 16S structure corresponding very closely to where Mrd1 binds in the 18S structure (Segerstolpe et al., manuscript in preparation). This indicates an evolutionary conservation of the function of RNA-binding proteins for the fold of the rRNA in this region, connecting the three major structural elements (the head, body and platform) of the small subunit.

### Functional implications of the modular architecture of Rbm19/Mrd1

The function of Rbm19/Mrd1 in ribosome biogenesis has been most extensively studied in yeast [9], [10]. Each of the five RBDs in Mrd1 (RBDs 1, 3, 4, 5 and 6, previously called RBDs1–5) is important for the overall function of the protein; RBD4 and 6 are essential, RBD1 very important and RBD5 the least important domain. For optimal function, all five RBDs need to be present.

The individual RBDs in Rbm19/Mrd1 are arranged in a defined order within the protein. In RNA-binding proteins, repeats of one or a few types of domains and a position specific conservation of the domains between orthologous proteins is often observed [21], [25]. In such proteins, different structural arrangements of the domains and a variety of permutations of the domains is essential for functional diversity and ability to interact with different substrates (for discussion, see [21]). *In vitro*, the individual RBDs in Mrd1 have low RNA-binding affinity, but a part of Mrd1 consisting of RBDs 1, 3 and 4 including linkers 1 and 3 has binding properties *in vitro* and *in vivo* similar to those of the full length Mrd1 (Segerstolpe et al., manuscript in preparation). In principle, the arrangement of specific RBDs within Rbm19/Mrd1 can thus be important for providing increased affinity and specificity for RNA-binding. It is furthermore possible that binding at different sites within the same RNA or within separate RNA molecules contributes to organizing specific RNA structures. This may include productive positioning of the RNA in relation to other proteins or RNAs.

The RBDs in Rbm19/Mrd1 are not only conserved as to sequence and position but the relative lengths of the linkers are also conserved, implying that the topological design of Rbm19/Mrd1 is functionally important. In other proteins, linker regions are important, influencing or directly contributing to RNA binding [21]. Short linkers can become structured upon RNA binding and participate in both RNA interactions and interactions between the neighbouring RBDs (for example [26]). Long linkers, theoretically longer than 50–60 residues [26], are expected to uncouple the RNA-binding affinity of neighbouring RBDs, provided that the linker does not bind to the RNA or fold into a structure that shortens its length.

The considerable length and apparent flexibility of linker 1 of Mrd1/Rbm19, possibly allowing independent movement of RBD1 in relation to the other RBDs, support the view that RBD1 binds independently from the other RBDs. Interallelic complementation studies of Mrd1 [9] support such a possibility. The partially structured linker 3 with a conserved length and sequence, located between RBD3 and RBD4 could indicate that these two RBDs interact with their target(s) in a coordinated manner. In accordance, RBD3 and RBD4 have to be present in the same molecule, suggesting that they are needed at the same step during pre-rRNA processing [9]. Linker 5 has a conserved short length and interallelic complementation suggests that the two RBDs connected by linker 5 may be functionally coupled [9], to potentially form a large binding surface.

### Evolutionary variations of the modular architecture of Rbm19/Mrd1

Apparently, RBD2 has been introduced during evolution of animals and Choanoflagellates, although we note that RBD2 is more diverged between species than the other RBDs. No experimental data is available that shows if RBD2 is functionally important or not, nor do we have any data indicating how RBD2 has been introduced or from where it originates. It is striking that RBD2 has been inserted in the long and disordered linker 1. This may reflect that the insertion did not disturb the topology of the rest of the protein or functional connections between RBDs 3 and 4 or between RBDs 5 and 6. The relatively close distance to RBD3 could indicate that the extra RBD contributes to the function of RBD3. It is plausible that Rbm19/Mrd1 has co-evolved with its substrate, the pre-rRNA, in which considerable variations in spacer sequences have arisen during eukaryotic evolution [27]. It is therefore possible that Rbm19 with its extra RBD co-evolved with a larger pre-rRNA.

Microsporidia have highly condensed genomes and they have Rbm19/Mrd1 proteins of exceptional small sizes [19]. Fungi generally have five RBDs (Fig. 2), but Microsporidia have only four RBDs. We therefore speculate that the Microsporidian homologues have been condensed and retain only four RBDs while all but one of the separating linkers have drastically reduced lengths. In agreement with such an interpretation, experimental findings for Mrd1 in *S. cerevisiae* have shown that at least two of the RBDs retained in Microsporidia (RBD1 and RBD6) are functionally either highly important or essential, whereas the lost RBD, presumably corresponds to the least important RBD in Mrd1, RBD5 [9]. In addition, the retained linker 3 is highly conserved (Fig. 7).

Rbm19/Mrd1 and thus presumably its function are maintained in these minimal intracellular parasitic organisms, underlining the essential nature of this protein. The Microsporidian genomes contain only about one third of the *S. cerevisiae* ORFs and over 85% of the proteins are smaller than the yeast orthologs [19]. The Microsporidium rDNA locus is prokaryotic-like and contains a 16S small subunit (SSU) and a 23S large subunit (LSU) rRNA, separated by only one internal transcribed spacer (ITS). The 5.8S rRNA is merged with the 5′ end of the 23S rRNA, similar to the prokaryotic rDNA organisation [28], [29], [30]. Although the SSU and LSU rRNAs are exceptionally small, (e.g. the SSU rRNA has a length of about 1300 nucleotides, as compared to about 1600 nucleotides in prokaryotes and about 1800 nucleotides in eukaryotes), they harbour typical eukaryotic ribosomal domains [28], [31]. Rbm19/Mrd1 is believed to function as a pre-rRNA structural modulator, possibly involved in forming the central pseudoknot structure, a conserved feature also for the Microsporidia small ribosomal subunit. Microsporidian Rbm19/Mrd1 may therefore have evolved in parallel with the reduced size of its ligand, the pre-ribosomal RNA.

### All RBDs except for RBD4 have conserved properties for RNA interaction

RBDs may contain extra β-strands, α-helices or an extended α1 [4]. Apart from an extra β-strand in loop 5 in RBDs 2, 3 and 5, no such special features are present in RBDs 1–6. The structures of several RBDs bound to short RNA substrates have been determined, showing that RNA-binding occurs in many different ways and that most elements within the RBD can be involved [5]. A single RBD can bind from two to eight nucleotides. The β-sheet is the primary binding platform, especially the central β1 and β3 strands. Here, four residues are frequently involved in binding a dinucleotide. Position 5 in RNP1 and position 2 in RNP2 mediate binding through base stacking with aromatic side chains, whereas hydrophobic interaction forms between an aromatic residue at position 3 in RNP1 and the sugar rings of the dinucleotide. A salt bridge between a positively charged residue in RNP1 position 1 and the phosphodiester group between the two nucleotides contributes as well [6]. One to four of these contacts may be present in a particular RBD and the base stacking interactions are the most common.

Out of the four common residues for RNA binding in RNP1 and RNP2, at least two of these are found in the RBDs 1, 3, 5 and 6 consensus sequences. RBD2 has in general diverged within the smaller group of more closely related organisms that contain this RBD. The presence of conserved common binding residues in RBDs 1, 3, 5 and 6 suggest that the β-sheet is involved in binding RNA, using one or more of the characteristic contacts. However, the differences in consensus sequences indicate that the individual RBDs use the β-sheet in different ways. Binding specificity for the central dinucleotide is often reached by contribution from non-conserved residues located within the β1 and β3 strands and the C-terminal of the β4 strand [4], [5]. Different conserved residues are present at such positions in for example RBDs 1, 5 and 6, suggesting that these residues contribute to specificity for RNA-binding for these RBDs. In Mrd1, replacement of the aromatic residues at RNP1 position 3 and 5 in RBD6 is lethal, supporting the hypothesis that these residues are involved in an essential interaction, presumably specific RNA-binding [9]. The corresponding replacements in the RNP1 motif in RBDs 1, 3 and 5 were functionally tolerated, showing that if stacking at these aromatic residues occurs, they are not required for the overall protein function.

Higher affinity and increased specificity in RNA-binding can be achieved by additional contacts. The β2 and β4 strands may contribute to RNA-binding (reviewed in [5], [6]). Conserved residues are present in both these strands, especially in β4 in RBDs 1, 3, 4, 5 and 6. Apart from the β-sheet, other elements can participate in RNA-binding. α1 may be involved using a conserved motif [32], while α2 as a rule is not participating. The loops can be important. Loops 1, 3 and 5 are often crucial. In specific RBDs, from one to all three of these loops may participate in RNA interaction (reviewed in [6]). In Rbm19/Mrd1, loops 1 and 5 are conserved in RBDs 3, 5 and 6 and may therefore be important for RNA interactions (Fig. 5C). Many RBDs (about 25% of all human RBDs) have an aromatic residue in loop 1 that is often important for RNA-binding [5]. Such a conserved aromatic residue is seen in the consensus for RBDs 3, 5 and 6 (Fig. 5A). Fox-1 and Rna15 form a binding pocket with a L/I/V-P-F/Y sequence in loop 1 and an arginine in loop 5, that can interact with RNA nucleotides 5′ of the dinucleotide, bound by the central β-sheet [33], [34]. RBDs 3 and 6 in human Rbm19 (Fig. 1B), both contain L/I/V-P-F/Y in loop 1 and an arginine in loop 5. By superimposing the structures for RBDs 3 and 6 with that of the Rna15 RBD (data not shown), these elements are overlapping to a large extent. The proline in loop 1 is not found in the consensus sequences for RBD 3 and 6, however the proline is conserved in 53% and 73% of the RBDs 3 and 6 sequences, respectively. Whether a similar binding pocket, as for the ones found in Rna15 and Fox-1, is formed upon RNA binding, needs to be experimentally determined.

Loop 5 is especially interesting in the consensus Rbm19/Mrd1 RBDs. In RBDs 2, 3 and 5 this loop includes short extra β-strands that can be involved in RNA-binding (for example [33]) and even in protein-protein interaction [35]. Conservation of loop 5 extends into the β4-strand in RBDs 1, 3, 5 and 6, indicating that in these RBDs, this region is important. In agreement with this hypothesis, we demonstrated experimentally that the conserved sequence in this region of RBD6 in Mrd1 is essential for the function of the protein. We do not yet know the putative interaction partner for this region.

The consensus sequence for RBD4 is different from those of the other RBDs in significant ways. In the β-sheet, aromatic residues are lacking both in the poorly conserved RNP1 and in the otherwise better conserved RNP2, suggesting that at least the RNP1 motif is not involved in RNA interaction. We found no species with an RBD4 in which aromatic residues are present both at position 3 and 5 in RNP1. Only three species have an aromatic residue in position 3 (*Leishmania braziliensis, Trypanosoma brucei brucei* and *Oryza sativa*) and no species has such a residue at position 5. In RNP2, only one species could be detected that has an aromatic residue at position 2 (*Paramecium tetraurelia*). RBD4 may still bind RNA because there are examples of RBDs that do not use the β-sheet for RNA interaction. In hnRNP F, so called quasi RBDs use loops 1, 3 and 5 for RNA interaction [36]. In this case, an aromatic residue in loop 1 and a β-strand in loop 5 are important. However, these features are not present in RBD4 of Rbm19/Mrd1. SF2/ASF contacts RNA at α1, β2 and loops 4 and 5 [32]. In RBD4 of Rbm19/Mrd1, conserved residues are essentially only seen in loop 5 and in particular in β4. Thereby the possibility remains that RBD4 may not contact RNA.

The early evolution and subsequent conservation of Rbm19/Mrd1 in all eukaryotes is not surprising in light of its essential role in synthesis of the small eukaryotic ribosome subunit. The conserved modular architecture and the conserved features of the individual RBDs provide guidance for experimental analyses of the mechanistic role of Rbm19/Mrd1 in the pre-ribosomal complex.

## Materials and Methods

### Identification of homologues

An initial Hidden Markov model (HMM) was created using the jackhmmer command in HMMER3 (version 3.0, http://hmmer.org), with MRD1_YEAST as seed sequence, and score of 200 as cutoff (parameter _incdomT). The final HMM (after seven iterations) identified 299 Rbm19/Mrd1 homologues (in 260 species) in UniprotKB version 2012 02. These were then aligned, using mafft-linsi [37] and the RDBs, as well as part of linker 3 (positions 496–581), were divided into seven separate domain alignments. The boundaries for the RBDs were as specified in UniProtKB for human RBD1, RBD2, RBD3, RBD5 and RBD6 (positions 2–79, 294–369, 402–480, 730–811, 832–912). For RBD4, the first strand in the 3D structure (PDB identifier 1WHX) exceeds the indicated start position (at position 587) by three residues; hence these were included in the alignment (positions 584–659). The domain alignments were cleaned from fragments and made non-redundant at 80% sequence identity. The resulting alignments were subsequently used as seeds when creating RBD- and linker 3-specific HMMs during an iterative process where homologues were successively added until no new sequences were found. Different cutoffs were tested starting at score ≥90 and decreasing in steps of five. The optimal cutoffs finally used, identifying as many true hits as possible without including any false hits, were set to 60 for RBD1, RBD4 and linker 3, 75 for RBD2 and RBD5, and 80 for RBD3 and RBD6. The stability of the final HMMs was tested using jack-knifing: One sequence at a time was left out, an HMM was created. If the sequence left out did not score higher than false sequences it would have been removed from the dataset. However, every sequence fulfilled this criterion and hence no one was removed. This process resulted in identification of 11 additional Rbm19/Mrd1 homologues (giving the total of 271 homologues), and also resulted in the final datasets used for creating the RBD and linker 3 motifs.

### Sequence motifs

Sequence motifs were calculated based on the 80% non-redundant final datasets for each RBD and linker 3. The datasets were of varying sizes: 108 – RBD1, 22 – RBD2, 102 – RBD3, 99 - RBD4, 98 – RBD5 and 79 – RBD6, and 87 – linker 3. In order for a position to be participating in a motif, at least 85% of the sequences were required to have a conserved residue in that position. A conserved residue was defined as having a BLOSUM62 score above zero towards the most common amino acid in that position. Also, in order for a specific amino acid residue to participate in a certain motif position, at least 10% of the sequences should have that residue.

### Phylogenetic tree analyses

We selected evolutionary representative full-length sequences, corresponding to Fig. 2 and one level further. Their respective RBDs were aligned using mafft-linsi [37], a phylogenetic tree was created using ClustalW [38] and displayed using HyperTree [39].

### Structural analyses

The known three-dimensional structures were analysed using ICM Browser (Molsoft LLC). Secondary structure assignments were calculated according to the DSSP algorithm [40]. Secondary structure predictions on human Rbm19 RBD1 and linker 3, as well as yeast and Microsporidian Mrd1, were made using Psipred [41]. Disorder predictions were made using OnD-CRF [42].

### Linker region analyses

The dataset consisted of every sequence identified as a homologue by the full length HMM as well as the RBD-specific HMMs. The sequences were aligned using mafft-linsi [37] and made non-redundant at 80% level, leaving 117 sequences for linker length analyses.

### Yeast strains and genetic manipulations

The RBD6 loop 5-β4 mutants were created by one-step PCR based gene integration [43]. A URA3 fragment with 5′ and 3′ ends corresponding to the RBD6 loop 5-β4 region of *MRD1* was made and genomically integrated into a myc tagged *MRD1* allele in a haploid cell containing the wt *MRD1* gene on a *TRP1* plasmid (pRS424/MRD1). The cells were then transformed with four different PCR fragments containing the appropriate mutations in the loop 5-β4-region and selected for growth on 5-fluoroorotic acid (5-FOA) plates. The mutants were verified by PCR and Western blot analysis and tested for growth on 5-fluroanthrannilic acid (5-FAA) plates.

To further analyse the functional consequences of the RBD6 loop 5-β4 mutants we created diploid strains with a conditional *GAL1-3HA-MRD1* allele and the RBD6 loop 5-β4 mutants by crossing the mutant strains with PLY178, carrying a *GAL1-3HA-MRD1* allele. Prior to crossing the mutant strains had undergone a plasmid shuffle from the wt *MRD1* gene on a *TRP1* plasmid (pRS424/*MRD1*) to a *URA3* plasmid with the wt *MRD1* gene (pS001). Diploid strains were selected on minus ura/minus trp plates and tested on 5-FOA supplemented with galactose to enable cells to loose the pS001 plasmid. Growth was then tested on YPD plates.

### RNA extraction and Northern blot analysis

The diploid cells were grown in glucose medium and depleted for Mrd1 during 0, 12 and 16 hours. Total RNA was extracted with the hot phenol method [44] and the RNA, 10 µg/lane, was separated on a 1% agarose-formaldehyde gel. Following transfer to zeta-probe membrane (Bio Rad) the filters were first methylene blue stained and thereafter hybridised with an oligonucleotide specific for the *S. cerevisiae* ITS1, between cleavage sites D and A_2_ (5′ GCTCTCATGCTCTTGCC 3′). The oligonucleotide was end-labelled with T4 polynucleotide kinase (New England Biolabs) and γ-^32^P ATP (Perkin Elmer) as described [10].
